# Spatial and temporal variability and distribution of emerging contaminants in South African freshwater and wastewater

**DOI:** 10.1038/s41598-025-33703-2

**Published:** 2026-02-13

**Authors:** Paki Israel Dikobe, Memory Tekere, Vhahangwele Masindi, Spyros Foteinis

**Affiliations:** 1 Department of Environmental Sciences, College of Agriculture and Environmental Sciences, University of South Africa (UNISA), P. O. Box 392, Florida, 1710 South Africa; 2 Water Research Centre, Smart Places Cluster, Council for Scientific and Industrial Research (CSIR), P.O Box 395, Pretoria, 0184 Pretoria, Meiring Naudé Rd, Brummeria South Africa; 3 Research Centre for Carbon Solutions, School of Engineering and Physical Sciences, Heriot-Watt University, Edinburgh, EH14 4AS UK

**Keywords:** Chemistry, Environmental sciences

## Abstract

Year-round variations of contaminants of emerging concern (CECs, also known as emerging contaminants) were examined in river water, dam water, and treated municipal wastewater in the South African setting. UPLC-MS/MS identified CECs belonging to different categories such as pesticides, licit and illicit recreational drugs, and particularly pharmaceuticals. Concentrations of up to 6 µg/L were identified but greatly varied spatially and temporally. Treated municipal wastewater was a harbinger for CECs (e.g., 6,055 ± 434 ng/L for efavirenz an HIV drug), while high CECs concentrations were also observed in river water (e.g., 3,228 ± 114 ng/L for acetaminophen). The high concentration for antiretroviral medication reflects the HIV/AIDS crisis in Sub-Saharan Africa and likely medicine misuse for illicit drug (whoonga/nyaope) manufacturing. Large intra-seasonal and intra-annual (seasonal) variations were observed (e.g., caffeine in dam water ranged from 73 ± 6 ng/L to 1,492 ± 30 ng/L), with overall high intra-seasonal and intra-annual variations (coefficient of variations up to 1.08 and 1.52, respectively) being observed. Individual risk quotients of up to 30 suggested high ecotoxicological risk potential. CECs pollution is apparent in South Africa, and likely across Sub-Saharan Africa and the Global South where similar problems persist, suggesting the need for effective wastewater treatment and policy intervention to curb CECs releases in freshwater.

**Supplementary Information:**

The online version contains supplementary material available at 10.1038/s41598-025-33703-2.

## Introduction

Fresh water is an increasingly important natural resource, given that over 99% of all water in the world is unusable by human, either due to its nature (e.g., saline water) or due to accessibility issues (e.g., most of the fresh water is stored in ice sheets in Antarctica and Greenland or as soil moisture)^1^. Yet, population growth and human activities acting on different scales greatly affect freshwater quality and availability^2,3^. For example, human activities such as agriculture, sewage, mining, and industry release different contaminants to the environment, such as microorganisms and bacteria^4^, (heavy) metals^5,6^, pesticides^7^, pharmaceutically active compounds (PhACs), personal care products (PCPs), and endocrine disrupting compounds (EDCs)^8^. These contaminants can affect freshwater ecosystems and render fresh water itself unfit for human consumption, while their removal might be particularly costly or even not feasible when using conventional treatment technologies. Of prime concern are organic compounds that up until recently remained undetected or believed not to be important pollutants, since recent knowledge has highlighted that even at trace levels these could be harmful to the environment and human health^9,10^. Therefore, ongoing effort is undertaken to regulate these organic compounds, which are known as contaminants of emerging concern (CECs)or emerging contaminants (ECs) ^2–4^.

CECs emanate from a wide array of chemical substances, such as the ones found in PhACs and PCPs^11–13^; industrial chemicals (e.g., inks and adhesives)^14,15^; and even from toxins excreted from cyanobacterial blooms whose growth is on the rise due to global warming and high nutrient releases in freshwater bodies^16^. Due to the ubiquitous use of these chemical substances the scale of the problem could be much larger than previously thought, since nearly 3,000 biologically active compounds emanating only from PhACs use have been identified in the environment^11^. Possible CECs pathways to fresh water include, but are not limited to, leaching of compounds from the soil^17–19^; direct emissions from wastewater treatment plants (WWTPs)^20–22^; storm drainage in rural and urban areas^23^; and atmospheric deposition^24–26^. As a result, CECs have already been identified at raw water that feeds drinking water plants, such as lakes^27,28^ and rivers^29,30^, with CECs main source being, by and large, the discharge of treated and particularly untreated sewage^31,32^.

Furthermore, CECs concentrations can greatly vary with time, since large seasonal variations have been reported in different water bodies such as rivers^33,34^. A main reason is that on certain times of the year, CECs concentrations could spike or be undetected due to, for example, high temperatures and/or dilution^35^. Therefore, to inform policy on CECs regulation, their concentrations in freshwater bodies and in municipal WWTPs effluents should be frequently monitored. Note that many WWTPs have not been designed for and do not (completely) remove CECs and therefore their effluents are considered CECs point sources^36^. In this regard, risk-based monitoring is important. However, it remains scarce in the Global South due to policy, cost, and technology restrains^37,38^.

In South Africa, and sub-Saharan Africa in general, CECs are grossly unregulated, but the problem has been acknowledged and efforts are underway to develop appropriate interventions, through policy making, to limit CECs exposure^39^. To underpin water quality management and to support effective policy making, knowledge on CECs occurrence and temporal variations is required. Even though previous research has focused on CECs occurrence in the South African setting, sampling was mainly restricted during summer and/or winter, as to represent the rainy and dry season respectively^35,40^. Therefore, a comprehensive monitoring and assessment study that sheds light on intra-seasonal, seasonal, and intra-annual variations is an important research gap that this study fills. For this reason, here, focus was placed on the greater area of Pretoria, using as a case study water collected from Roodeplaat Dam, where activities in its catchment include agriculture, industry, urban run-offs, and sewage^41^. The dam feeds a local drinking water plant, and thus it was monitored on a monthly basis. Furthermore, spot measurements were also taken from river water (Mamelodi River, well downstream from the examined dam) and from the effluents of two municipal WWTPs, since municipal WWTPs are known to be associated with elevated CECs levels in the South African setting^36 and further afield31 ^.

Overall, this work provides insight about the nature and extent of CECs occurrence in South African water bodies and particularly about their temporal variation in a typical catchment basin. This knowledge could be instrumental for sub-Saharan Africa, where similar plights are encountered, such as the human immunodeficiency virus (HIV) / acquired immunodeficiency syndrome (AIDS) crisis, given that freshwater and wastewater can provide important information about the geographic distribution and prevalence of HIV in local communities. Note that globally, the majority of the people that live with HIV reside in Sub-Saharan Africa^42^. It can also provide insight for other areas across the developing world, where similar practices such as pesticide overuse and/or drug abuse are followed.

## Materials and methods

### Contaminants of emerging concern under study

Here, focus was placed on CECs that have been previously identified in wastewater and fresh water in South Africa^35,40^. Specifically, 21 CECs that cover different categories were examined, including PhACs (e.g., antibiotic and antiretroviral medication), PCPs (e.g., caffeine)^8^, and EDCs (e.g., atrazine, a herbicide that has been banned in the European Union^7^ but is applied in South Africa during the dry season^43,44^. A detail list of the CECs under study and background information for each one is given in (Table 1).

The examined CECs not only cover compounds with high-risk potential for human and the environment, but also provide insight about agricultural practices and mainly medicine and illicit drugs usage in the catchment area, and in South Africa and sub-Sahara Africa in general. This knowledge is vital for preventing and mitigating risks related to ecosystem and human exposure to CECs, but it can also help in assessing community health and inform about unhealthy lifestyles (e.g., medication abuse and illicit drugs usage). For example, the concentrations of emtricitabine and efavirenz, both antiretroviral medications, could provide insight about the HIV prevalence in the catchment area and likely illicit drug use.

**Table 1:** The examined CECs along with background and common applications.

Categories	Compound	Acronym	Background, parent compound, and use
Stimulant	Methamphetamine 1,7 Dimethylxanthine 3,4-Methylenedioxy-methamphetamine Cocaine Benzoylecgonine Caffeine	MET 1,7 DXN MDMA COC BZE CAF	Recreational (illicit) drug Coffee/tea (metabolite of caffeine) Illicit drug (ecstasy) Illicit drug Illicit drug (cocaine metabolite) Coffee/tea
Analgesic/Anti-inflammatory	Acetaminophen Diclofenac Naproxen	ACT DCF NAP	Painkiller and fever treatment Nonsteroidal anti-inflammatory drug (NSAID) NSAID
Antibiotic	Sulfamethoxazole Trimethoprim	SMX TMP	Cure bacterial infections Cure bacterial infections
Antiretroviral	Emtricitabine Efavirenz	FTC EVZ	HIV treatment HIV treatment
Narcotic analgesic	Tramadol Codeine	TMD COD	Opioid pain medication Mild opioid pain reliever
Antidepressant	Venlafaxine	VFX	Serotonin and norepinephrine reuptake inhibitors (SNRIs)
Piperazine	Cetirizine	CTZ	Non-drowsy antihistamine
Antiepileptic	Carbamazepine	CBZ	Anticonvulsant medication
Industrial inhibitor	Benzotriazole	BTA	Corrosion inhibitor for metals (copper)
Agriculture	Atrazine	ATZ	Chlorinated herbicide
Sedative	Methaqualone	MTQ	Hypnotic sedative

### Sampling locations and sampling frequency

All samples were collected from the greater area of the City of Tshwane Metropolitan Municipality, simply referred to as the City of Tshwane, in the Gauteng Province, South Africa. The catchment area is predominated by agricultural activities, industrial processes, urban run-offs, and treated and untreated sewage effluents, which are typical for the South African setting^41^ and eventually introduce CECs to aquatic ecosystems. Specifically, residential areas and informal settlements are found in the catchment area, while farming, wastewater treatment, industrial activities, and mining do also take place^41,43,45^. Illegal discharge and disposal of substances is amongst the topical issues of concern in the region, hence greatly contributing to the high levels of pollution reported in the region and across South Africa^36,43,45,46^.

Due to limitations in technology (mainly equipment availability for CECs quantification) and cost barriers it was not possible to frequently monitor different water matrices from within the examined catchment area. For this reason, focus was placed on assessing the quality of the raw water (i.e., dam water) that feeds a local drinking water treatment plant, from where a comprehensive year-round time series (comprising monthly measurements) was acquired. It should be noted that this water treatment plant, as others in South Africa, is based on a conventional treatment train, mainly aiming at removing pathogens, comprising coagulation, sedimentation, (sand) filtration, and chlorination^43,44^. A detail analysis of how CECs in raw water pass through each treatment stage of this specific plant can be found in Dikobe et al., (2024)^44^. As such, CECs occurrence in raw water can greatly define their final concentrations in the final drinking water, since these are known to persist in conventional drinking water treatment^4^.

**Fig. 1:**
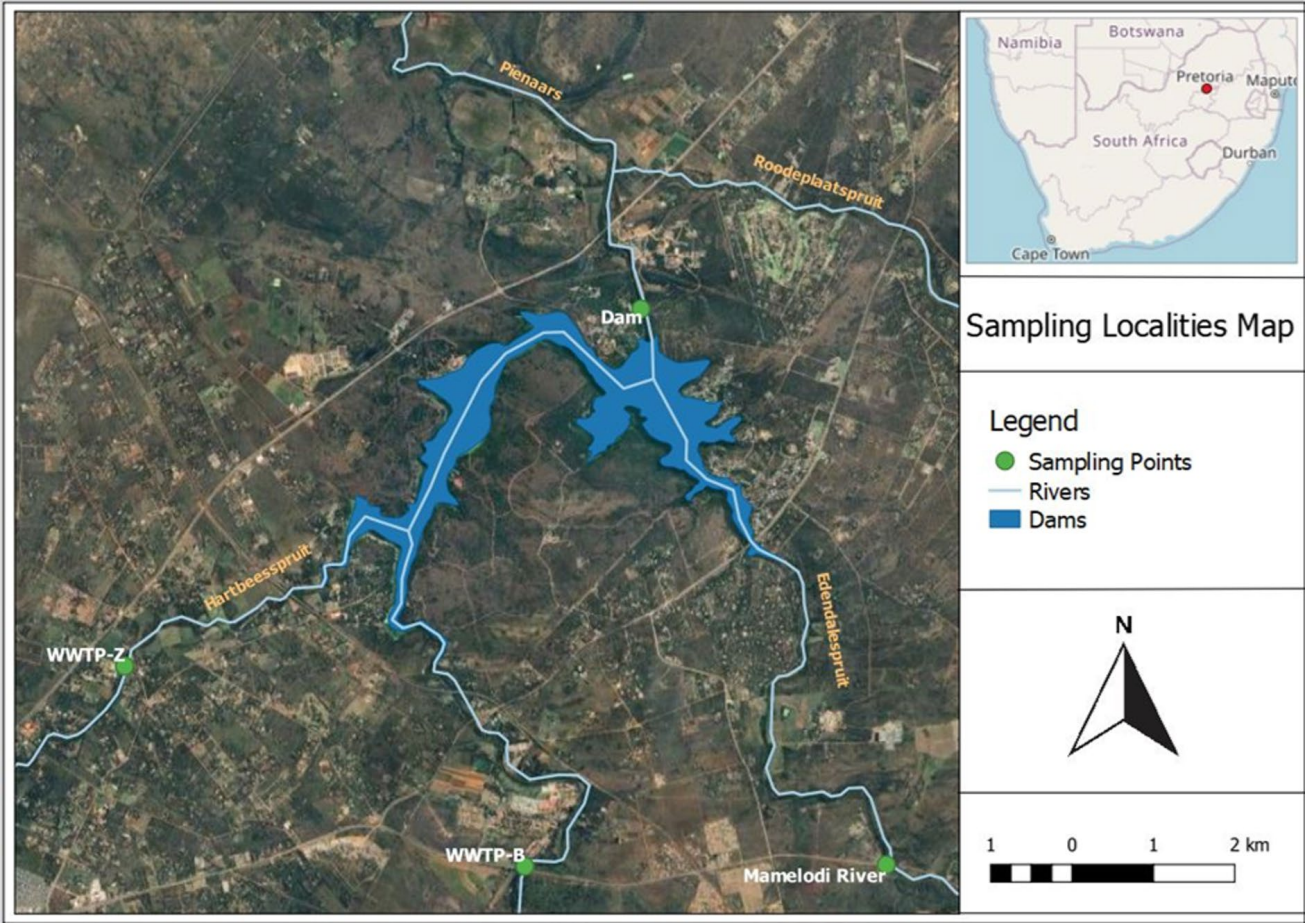
The map of the study area and locations from where the examined samples were collected, as produced using QGIS (version 3.28.4).

The dam water time series was complemented by single measurements from two different water matrices from within the catchment area, i.e., from the treated effluents of two (2) municipal WWTPs, i.e., WWTP-B and WWTP-Z (S25^o^39.379’ E28^o^18.501’) and from Mamelodi River/stream (Fig. 1**)**. Dam water samples were collected during the reference period 17th of January to the 2nd December 2022. Specifically, every month a field survey took place, and dam water was collected. In addition, during the field survey of the 17th of November 2022, additional samples were also collected from the two municipal WWTPs and from Mamelodi River. All samples were collected using the grab sampling technique. For consistency, the sampling was carried out in the morning of each sampling date. In total 15 samples were collected, i.e., twelve monthly samples from dam water, one sample from river water, and two samples from the two different municipal WWTPs. As such, the collected samples provide a snapshot of the CECs concentrations on the date and time (morning) of collection.

### Sample collection and pre-treatment

Samples were hand grabbed using 500 mL sterile wide-mouth high-density polyethylene (HDPE) bottles. Before sampling, the bottles were rinsed twice using the sampling water and then immersed at about 5 to 10 cm beneath the water surface until they were filled up and then tightly sealed. The samples were then stored in a cool box for transportation, and upon arrival at the laboratory they were stored overnight at 4 °C and analysed the next day.

For CECs identification and quantification offline solid-phase extraction (SPE), followed by ultra-performance liquid chromatography (UPLC) with tandem mass spectrometry (MS/MS) was employed^47,48^. Due to CECs nature and very low concentrations, a hydrophilic-lipophilic balanced (HLB) extraction cartridge (sorbent) was used as the extraction phase. The extraction process was assisted and sped up using a 12-ports vacuum manifold (Supelco, VISIPREP™). In more detail, each sample (500 mL total volume) was split into 100 ml aliquots, which were then fortified (spiked) with 0.05 mL of a 1 mg/L internal multi-component (all the examined CECs) standard mixture and centrifuged (5,000 rpm for 15 min at 4 ˚C) to separate the solid particulate matter from the liquid phase and prevent clogging during pre-filtration^20^. The concentration of the internal multi-component standard was accounted for in CECs quantification, i.e., this was subtracted from the obtained results. A multi-component standard of the mass-labelled sample with all target species was used to deduce calibration curves of species, wherein given peaks within the spectra enabled the identification and quantification of compounds or elements present in the sample which ultimately gave the concentrations of the target species^49^. The supernatant was then filtered under vacuum phase using pre-prepared 0.45 μm pore size glass fibre filters (GF/F, Whatman, CAT 1825-045), i.e., the filter papers had been rinsed with 2 mL of methanol and flushed with MilliQ water to remove any particles that might be suspended on the fine glass fibres.

In SPE, extractions of the selected CECs were performed using the Oasis^®^ HLB cartridges (3 cc, 60 mg; Waters), which were conditioned with 2 mL of UPLC grade methanol (MeOH) followed by rinsing with 2 mL of ultrapure water. The liquid samples were then filtered (flow rate 5 mL/min) through the cartridges using the vacuum manifold and the cartridges were left to dry under a gentle stream of nitrogen. Analytes were eluted with 7 mL of MeOH at a flow rate of 1 mL/min. The eluate was subsequently concentrated to 2 mL under a gentle stream of nitrogen and was further centrifuged for 25 min prior analysis.

### CECs analysis

UPLC (Waters ACQUITY) was used to acquire the chromatograms of the pre-treated samples. Different gradient elution methods were developed for the same column and solvents were prepared to quantify the targeted CECs. In more detail, the mobile phase-A (ultrapure water containing 0.1% formic acid) and mobile phase-B (100% UPLC grade methanol) were optimized using linear gradient elution, with an initial composition of 100% mobile phase-A, maintained for 0.2 min, reduced to 10% mobile phase-A over 6.8 min, and finally to 0% mobile phase-A for 0.1 min (100% mobile phase-B). This was then returned to 100% mobile phase-A for 0.4 min and maintained for 2.5 min for re-equilibration (total run time 10 min). This method used a reversed-phase BEH C18 column (1.7 μm pore size, 2.1 × 100 mm, Waters ACQUITY), fitted with a 0.2 μm in-line column filter. Other considered parameters include column temperature (50 °C), sample injection volume (2 mL), run time (10 min), and mobile phase flow rate (0.4 mL/min). Target CECs identification and quantification was achieved using a tandem quadrupole mass spectrometer (MS/MS) (Xevo TQ-MS), making use of the Step Wave off-axis ion source technology. To obtain an adequate number of points, which are required for analyte confirmation and quantification, mass acquisition was performed using the multiple reaction monitoring method.

### Certified standards

Analytical grade reagents were used throughout. For CECs quantification 1000 mg/L standards, containing the targeted CECs, were purchased from Merck–Supelco, S.A. and used to prepare calibration and quality control standards. High purity nitrogen (99.50%) and argon (99.99%) gases were purchased from Air Liquide S.A. Furthermore, 20% formic acid and 100% UPLC grade methanol were purchased from Sigma-Aldrich S.A., whereas ultrapure water (Milli-Q Water Purification System Sigma-Aldrich S.A) was used where required.

### Accuracy

Care was taken to prevent blank contamination from the analytical pieces of equipment, organic solvents, glassware, and personnel. The prepared blank samples from ultrapure water were extracted and analysed, along with the actual samples and the quality control standards, in order to monitor potential laboratory contamination for the CECs under study. Methanol blanks were employed to monitor cross contamination and instrument contamination. Analytes of interest were characterized using signal noise and chromatographic peak area and height, whilst peak area was used to measure the optimal signal intensities for quantification.

The method was validated as summarised in Table 2, and the validity of the generated results was ascertained via the analysis of the generated multi-element quality control standards (i.e., 10, 100, 500 µg/L) and duplicates during the quantification process. During validation, percentage recoveries were between 90 and 110% and accuracy was approximately 100%. The reproducibility and repeatability were determined by comparing the obtained concentrations of the same raw samples in consecutive days and within short interval between two analyses. A high level of confidence for the obtained results was achieved, since the calibration curves (ten concentration levels (1 µg/L to 500 µg/L) in the same solvent as the re-constituted water samples (MeOH)) yielded coefficients of determination (R^2^) in the range 0.990–0.999 for the selected CECs. Precision was determined using replicate (5) injections of each calibration standard.

The obtained results for selectivity were evaluated by comparing the retention time of the peaks with those of the standard solutions. Simultaneously, the identification of the analytes was confirmed by comparing the corresponding spectra of the peaks in the chromatograms of the sample with the one of the standard solutions. The integration of the analyte standard curves with the water sample concentrations was determined using the TargetLynx software (Version 4.1, Waters).

### Limit of detection and quantification

The limit of detection (LOD) and quantification (LOQ) for the targeted CECs were estimated using a signal to noise (S/N) ratio of 3 and 10, respectively. The obtained LOD and LOQ values are listed in Table 2, where, as can be seen, overall, very low limits were obtained for both.

### Ecotoxicological risk

To gain insight about the potential ecotoxicological risks of the targeted CECs, the risk quotient (RQ) approach was employed, whereby the measured environmental concentration (MEC) of each CEC was normalized using the predicted no-effect concentration (PNEC), as obtained from the NORMAN ecotoxicology database^50,51^. Based on previous research, the highest concentration, as obtained from the year-round measurement campaign, for each CEC per site, was used as the MEC value^52^. Classification of risk for CECs was assigned as follows: RQ < 0.1 = insignificant risk; 0.1–1.0 = low risk; 1.0–10 = medium risk; and > 10 = high risk^21,53^.

**Table 2:** Results for the accuracy and linearity of the employed method.

CEC	Quantification ion (m/z)	Linearity range (µg/L)	Coefficient Value	LOD (µg/L)	LOQ (µg/L)	Percentage Recovery (%)
ACT	152.071	1–500	0.997–0.999	0.291	0.882	98.00
ATZ	214.010	1–500	0.997–0.998	0.061	0.184	99.00
BTA	147.063	1–500	0.997–0.999	0.015	0.046	97.00
BZE	83.010	1–500	0.990–0.993	0.010	0.032	99.00
CAF	195.088	1–500	0.990–0.993	0.530	1.607	98.00
CBZ	237.102	1–500	0.997–0.998	0.015	0.046	99.00
COC	83.203	1–500	0.997–0.999	0.005	0.016	99.00
COD	300.301	1–500	0.990–0.992	0.005	0.016	98.00
CTZ	389.154	1–500	0.997–0.999	0.005	0.016	97.00
DCF	296.024	1–500	0.990–0.996	0.061	0.184	98.00
EVZ	316.035	1–500	0.997–0.998	0.059	0.179	99.00
FTC	248.050	1–500	0.997–0.999	0.010	0.032	98.00
MDMA	194.201	1–500	0.997–0.999	0.010	0.032	99.00
MET	119.010	1–500	0.997–0.999	0.005	0.016	98.00
MTQ	235.101	1–500	0.997–0.999	0.005	0.016	99.00
NAP	231.102	1–500	0.995–0.997	0.565	1.711	98.00
SMX	254.059	1–500	0.997–0.999	0.035	0.106	98.00
TMD	264.196	1–500	0.997–0.998	0.010	0.032	97.00
TMP	291.145	1–500	0.997–0.999	0.006	0.019	99.00
VFX	278.212	1–500	0.997–0.998	0.005	0.016	98.00
1,7 DXN	181.072	1–500	0.990–0.992	0.330	1.000	99.0

### Variability

Finally, for each CEC the intra-seasonal and intra-annual (seasonal) variabilities for the dam water samples were estimated by using the coefficient of variation (COV). This is a common index for assessing temporal variability and can be estimated by dividing the standard deviation (σ) of the corresponding time series with its mean (µ)^54^, as shown in Eq. (1).

1 $$\:COV=\frac{\sigma\:}{{\upmu\:}}$$

Here, summer season corresponds to: December, January, February; autumn season: March, April, May; winter season: June, July, August; and spring season: September, October, November. In general, COV values lower than unity (1) suggest low-variance, whereas values higher than unity suggest high-variance. Here, the monthly mean values of each examined CEC were used to estimate the COV values for the intra-annual (seasonal) variability. Note that the results presented herein are indicative and could mainly reflect CECs patterns in this specific catchment area. Denser time series, such as the ones referring to daily or weekly measurements, could provide more insight into CECs variations.

## Results and discussion

### CECs occurrence and variation in dam water

In Table 3 the measured CECs concentrations are shown, along with the estimated standard deviations (σ). Out of the 21 examined CECs, 19 were detected in dam water since cocaine and ecstasy (MDMA) were not identified in dam water. This is also the case for codeine and diclofenac for winter and spring seasons, whereas in spring acetaminophen was detected, but was below the LOQ. The antiretroviral drug efavirenz had the highest levels in all seasons, apart from autumn where 1,7 Dimethylxanthine (caffeine metabolite) had the highest concentration. Overall, different seasonal patterns were observed for the CECs that were above the LOQ. Specifically, in summer season efavirenz was the CEC with the highest concentration (912 ± 220 ng/L), followed by emtricitabine (328 ± 342 ng/L), tramadol (298 ± 138 ng/L), atrazine (250 ± 38 ng/L), and carbamazepine (129 ± 13 ng/L). In autumn season 1,7 Dimethylxanthine (592 ± 235 ng/L) was closely followed by efavirenz (564 ± 43 ng/L) and emtricitabine (555 ± 125 ng/L), and then caffeine (287 ± 168 ng/L) and atrazine (147 ± 22 ng/L). In winter season efavirenz was again the CEC with the highest concentration (809 ± 206 ng/L), followed by 1,7 Dimethylxanthine (574 ± 85 ng/L), atrazine (247 ± 37 ng/L), emtricitabine (244 ± 225 ng/L carbamazepine (128 ± 25 ng/L), tramadol (129 ± 58 ng/L), and caffeine (106 ± 50 ng/L). Finally, in spring season efavirenz was, by and large, the CEC with the highest concentration (2205 ± 636 ng/L), followed by emtricitabine (402 ± 139 ng/L), 1,7 Dimethylxanthine (351 ± 134 ng/L), atrazine (264 ± 63 ng/L), tramadol (251 ± 17 ng/L), sulfamethoxazole (143 ± 50 ng/L), and carbamazepine (143 ± 12 ng/L) (Table 3).

For the year-round reference period (intra-annual or average values), a similar pattern was identified with efavirenz being the CEC with the highest concentration, followed by 1,7 emtricitabine, atrazine, tramadol, and caffeine. The high concentrations for efavirenz, emtricitabine, and tramadol could suggest direct disposal of PhACs and drugs to raw water, but more likely, given the high levels of caffeine and its metabolite (1,7 Dimethylxanthine), that untreated or poorly treated municipal wastewater is released to raw water and end ups in the dam^55–57^. Furthermore, the identified levels of atrazine and benzotriazole could suggest that agricultural and other industrial activities also take place in the catchment area, which eventually release CECs in receiving water bodies, in this case the river/stream water that is collected in the dam^43,44^.

Relatively high temporal variabilities were also observed across the examined CECs, with COV values as high as 2.15 being observed (Table 3). In more detail, intra-seasonal variability was in the range 0.04 (naproxen) to 1.73 (acetaminophen and emtricitabine), with many CECs yielding overall high COV intra-seasonal values but only acetaminophen, caffeine, codeine, and diclofenac yielding values of unity and above, i.e., exhibiting high temporal variance. However, for diclofenac and codeine this could be, at least partially, traced back to their consistent relatively low concentrations. The seasonal variability appears to be lower than the intra-seasonal, with the variability during spring and autumn seasons being lower than summer and winter seasons. Regarding the intra-annual variability this appear to be on par with the intra-seasonal variability, and as such is higher than the seasonal variability since the concentration of each examined CECs greatly vary between months. The observed temporal variations could be possibly traced back to the effect of rainfall, since a high spike in CECs was identified between the end of South Africa’s dry season (winter) and the beginning of the wet season (spring). We hypothesize that the observed spikes could possibly be traced back to the increased runoffs during the first rains of the wet season. However, it should be noted that the presented results are only indicative of the possible CECs variations within the examined catchment area and longer-term measurements are needed to shed further light on the identified seasonal, intra-seasonal, and intra-annual variations.

However, result imply that, at minimum, CECs in water should be monitored at least at a seasonal level, i.e., one measurement per season. Yet, the high seasonal and intra-annual variability imply that denser time series, likely weekly or even daily, could be required to accurately capture an accurate baseline and identify CECs temporal variations. Such detail datasets could also provide meaningful information about the local community’s health status and the extent of the treatment that drinking water requires to remove CECs. Note that the examined dam water feeds a water treatment plant and many CECs could persist in the environment and also through drinking water treatment^4^. For example, EVZ, which exhibited overall high concentrations, is nonpolar and has low water solubility suggesting that more advance treatment such as activated carbon adsorption is required for its removal^58^. Yet, such treatment is not used in the local water treatment plant, nor is typical in South Africa and across the developing world (Global South).

**Table 3:** Levels (ng/L ± σ) of selected CECs along with their intra-seasonal (summer, autumn, winter, spring seasons) and intra-annual (seasonal) variations in dam water.

ECs	Summer		Autumn		Winter		Spring		Range	Intra-seasonal		Intra-annual	
	Mean ± σ	COV	Mean ± σ	COV	Mean ± σ	COV	Mean ± σ	COV		Mean ± σ	COV	Mean ± σ	COV
ACT	15.50±16.02	1.03	50.42±31.52	0.63	4.75±8.23	1.73	<LOQ	-	0 -72	17.67±22.78	1.29	17.67±25.77	1.46
ATZ	250.00±38.57	0.15	147.00±22.34	0.15	246.58±37.40	0.15	264.25±63.45	0.24	129-321	226.96±53.85	0.24	226.96±60.99	0.27
BTA	76.50±22.59	0.30	39.42±4.50	0.11	59.00±1.00	0.02	69.83±15.46	0.22	35-96.5	61.19±16.21	0.26	61.19±18.84	0.31
BZE	11.17±2.02	0.18	6.33±5.51	0.87	14.50±2.18	0.15	6.42±5.59	0.87	0-17	9.60±3.97	0.41	9.60±5.07	0.53
CAF	75.08±34.76	0.46	511.75±223.27	0.44	105.83±49.64	0.47	83.83±41.97	0.50	35-786.3	194.13±212.14	1.09	194.13±216.50	1.12
CBZ	129.42±12.76	0.10	91.50±14.24	0.16	127.75±24.91	0.20	142.83±11.77	0.08	79-155	122.88±21.98	0.18	122.88±24.49	0.20
COC	ND*	-	ND	-	ND	-	ND	-	-	-	-	-	-
COD	14.33±24.83	1.73	92.33±92.50	1.00	ND	-	ND	-	0-185	26.67±44.30	1.66	26.67±57.21	2.15
CTZ	32.58±10.88	0.33	18.17±3.25	0.18	23.00±15.87	0.69	61.08±7.84	0.13	5-70	33.71±19.21	0.57	33.71±19.55	0.58
DCF	8.42±7.42	0.88	12.33±10.69	0.87	ND	-	ND	-	-	5.19±6.20	1.20	5.19±7.89	1.52
EVZ	912.50±219.77	0.24	564.25±42.90	0.08	733.75±254.68	0.35	2205.25±636.36	0.29	517-2940	1103.94±747.85	0.68	1103.94±743.05	0.67
FTC	328.17±341.46	1.04	555.08±124.88	0.22	148.00±256.34	1.73	402.25±138.74	0.34	0-685	358.38±169.10	0.47	358.38±250.76	0.70
MDMA	ND		ND	-	ND	-	ND	-	-	-	-	-	-
MET	6.75±3.38	0.50	16.33±4.93	0.30	8.83±5.20	0.59	2.33±2.52	1.08	0-22	8.56±5.85	0.68	8.56±6.37	0.74
MTQ	82.42±36.05	0.44	65.67±8.14	0.12	70.25±1.30	0.02	70.25±26.71	0.38	51-124	72.15±7.18	0.10	72.15±20.51	0.28
NAP	23.17±1.04	0.04	31.25±5.17	0.17	15.33±13.43	0.88	32.83±13.97	0.43	0-48	25.65±8.07	0.31	25.65±11.25	0.44
SMX	96.67±14.50	0.15	96.58±63.62	0.66	94.75±86.42	0.91	143.25±49.73	0.35	0-191	107.81±23.64	0.22	107.81±55.13	0.51
TMD	297.42±137.59	0.46	80.17±16.00	0.20	129.08±57.87	0.45	251.42±17.13	0.07	69-432	189.52±101.79	0.54	189.52±112.38	0.59
TMP	35.50±15.16	0.43	52.75±23.71	0.45	67.83±31.08	0.46	80.42±40.38	0.50	26.5-121	59.13±19.39	0.33	59.13±30.39	0.51
VFX	30.00±18.68	0.62	6.08±2.79	0.46	14.00±9.17	0.65	36.17±12.29	0.34	4-50	21.56±13.92	0.65	21.56±16.31	0.76
1,7 DXN	96.25±71.40	0.74	591.75±235.00	0.40	573.92±84.86	0.15	351.25±134.09	0.38	35.8-768.3	403.29±232.10	0.58	403.29±244.18	0.61

*ND = Not detected.

*ND = Not detected.

### CECs occurrence in river water and treated municipal wastewater

The CECs concentrations, which correspond to the 17 November 2022 field survey, in river/stream and treated municipal wastewater are shown in Table 4. For context, the CECs concentrations in dam water are also given. In river water, low concentrations for some PhACs were identified (e.g., codeine was not detected while methaqualone’s average concentration was just 5.3 ± 0.3 ng/L), but very high for others (e.g., acetaminophen (3,227.8 ± 113.5 ng/L), efavirenz (2,210.2 ± 248.5 ng/L), and emtricitabine (3790.7 ± 170.8 ng/L)). The spike value for emtricitabine (nearly twice than efavirenz concentration) in river water is inconsistent with the measured values in dam water, where efavirenz concentration (range 517-2,940 ng/L) was consistently higher than emtricitabine concentration (0–685 ng/L). This could trace back to pharmacodynamics, metabolism, and other interactions (e.g., photolysis). For example, when released to the environment emtricitabine will slowly degrade whereas efavirenz is less degradable and therefore is considered as potentially persistent^59^. Furthermore, the very high concentrations of HIV drugs (efavirenz and emtricitabine) in fresh water reflect the current AIDS crisis in South Africa and Sub-Saharan Africa^42^ and could also allude to possible drug misuse. For example, in HIV medication different ratios of HIV drugs are employed to obtain the highest degree of synergy between the active ingredients of each drug, such as a ratio of 3:1 for EVZ (600 mg) to FTC (200 mg) when used for HIV-1 treatment in adults^60^. However, the measurements of efavirenz and emtricitabine (Table 4) appear not to follow this or another specific pattern, which when accounting for pharmacodynamics, metabolism, and other interactions could imply a specific ratio between those HIV drugs. As such, this could suggest that the reason behind the very high concentrations of these CECs could not only trace back to the treatment of HIV infection, a known problem across Sub-Saharan Africa^42^, but also to antiretroviral abuse^61,62^. Furthermore, even though a comparison with the dam water cannot be direct, higher CECs concentrations, such as emtricitabine, acetaminophen, and caffeine, were identified, likely tracing back to pharmacodynamics, metabolism, and other interactions.

Regarding the occurrence of CECs in treated municipal wastewater, it was identified that in both WWTPs most CECs yielded high concentrations, and particularly efavirenz (6055.3 ± 433.8 ng/L) (Table 4). However, CECs concentrations greatly varied between the examined WWTPs, suggesting a high spatial variability in wastewater matrices, since both treatment plants are nearby and follow similar treatment practices. For example, the concentration of atrazine^63^, a widely used herbicide in South Africa^43^, was two orders of magnitude higher in WWTP-Z (2863.8 ± 60.5 ng/L) than in WWTP-B (48.8 ± 3.2 ng/L), but this could well be attributed to typical variations when measuring CECs. Furthermore, in WWTP-Z the mean concentration of EVZ was nearly thrice the one of WWTP-B and in both cases high FTC concentrations (1805.5 ± 53.0 ng/L ng/L in WWTP-B and 1515.3 ± 20.9 ng/L in WWTP-Z) were identified (Table 4). This could suggest that some of the population in the catchment areas of the examined WWTPs is receiving antiretroviral therapy and/or possibly allude to increased antiretroviral medication abuse for illicit drug (whoonga) manufacturing and consumption^64,65^. The low benzoylecgonine (13.8 ± 1.1 ng/L ng/L in WWTP-B and 2.2 ± 0.4 ng/L in WWTP-Z) concentrations could possibly explain why cocaine was not detected. Specifically, after use only a small amount (1–9%) of the cocaine is excreted unchanged and most (35–54%) is metabolized and excreted as benzoylecgonine^36^. The concentrations of other CECs were also elevated in the WWTPs effluents (Table 4), suggesting that such effluents could be a harbinger for CECs in South Africa and possibly in sub-Saharan Africa where similar production and consumption practices, population health, and/or similar or even more sub-par wastewater treatment practices are followed.

Overall, results suggest that catchment activities (e.g., use of herbicides) and local population health (e.g., people affected by HIV) and behavior (e.g., illicit drug manufacturing and consumption) could act as point sources for the diffuse of CECs in water. Therefore, CECs monitoring in water could provide insight about the health and activities within the catchment area and also about the degree of treatment that is required to remove CECs from raw water. This information is indispensable and can inform policy making for CECs regulation in water in South Africa and across the developing world.

**Table 4:** Levels (ng/L ± σ) of selected CECs in dam and river/stream water and in treated municipal wastewater effluents during the 17th of November 2022 field survey.

CEC	Dam water	River water	WWTP-B	WWTP-Z
ACT	< LOQ	3227.8 ± 113.5	186.8 ± 3.9	< LOQ
ATZ	195.8 ± 13.1	12.7 ± 0.3	48.8 ± 3.2	2863.8 ± 60.5
BTA	79.5 ± 9.9	8.8 ± 0.3	50.5 ± 1.4	104.8 ± 2.5
BZE	10.3 ± 0.4	13.5 ± 0	13.8 ± 1.1	2.2 ± 0.4
CAF	73.5 ± 6.4	1491.5 ± 29.7	1038.0 ± 1.4	273.5 ± 7.1
CBZ	131.5 ± 2.1	64.3 ± 1.1	108.0 ± 7.1	237.8 ± 7.4
COC	ND*	ND	ND	ND
COD	ND	ND	ND	ND
CTZ	55.3 ± 1.8	14.3 ± 1.0	36.0 ± 0.7	195.3 ± 2.5
DCF	< LOQ	67.2 ± 0.3	87.0 ± 0.7	98.3 ± 10.3
EVZ	1845.8 ± 101.5	2210.2 ± 248.5	2022.0 ± 3.5	6055.3 ± 433.8
FTC	418.8 ± 1.1	3790.7 ± 170.8	1805.5 ± 53.0	1515.3 ± 20.9
MDMA	ND	ND	ND	ND
MET	5.0 ± 0.7	82.2 ± 4.6	12.8 ± 0.4	51.5 ± 0.0
MTQ	100.8 ± 3.2	5.3 ± 0.3	66.0 ± 5.0	111.0 ± 2.8
NAP	20.5 ± 0.7	67.75 ± 2.5	90.8 ± 2.5	84.8 ± 4.6
SMX	91.8 ± 3.2	220 ± 9.9	153.8 ± 1.1	84.5 ± 0.7
TMD	268.3 ± 37.8	130.5 ± 5.7	305.5 ± 1.4	481.0 ± 11.3
TMP	40.3 ± 6.0	149.2 ± 0.3	218.8 ± 27.2	104.0 ± 6.4
VFX	26.5 ± 3.5	23 ± 1.0	14.0 ± 0.7	149.3 ± 3.2
1,7 DXN	248.8 ± 19.5	663.2 ± 49.8	717.0 ± 36.8	218.3 ± 5.3

*ND = Not detected and < LOQ = Below the Limit of Quantification.

### Percentage distribution of CECs in surface and treated wastewater and comparison with other studies

**Fig. 2:**
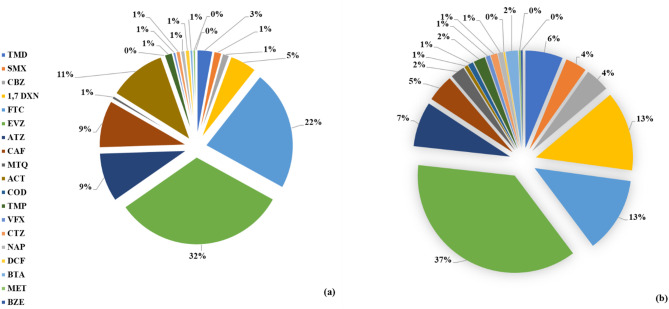
Percentage distribution of CECs in **a**) wastewater effluents and **b**) raw (dam and river) water. Note that with 0% the insignificant contributions (< 0.1%) are reported.

The CECs percentage distributions, based on their total concentrations in wastewater and surface water (river/stream and dam water) are summarised in Fig. 2. Efavirenz (EVZ) and emtricitabine (FTC) were the main contributors, 32% and 22% respectively, in wastewater, followed by acetaminophen (ACT) (11%), atrazine (ATZ), caffeine (CAF) (9%), 1,7 dimethylxanthine (1,7 DXN) (5%), and tramadol (TMD) (3%). All other CECs contributed 1% of less; while COD, BZE, and MET contribution of was insignificant (< 0.1%) to the overall CECs percentage distribution (Fig. 2a). In surface water, efavirenz had again the highest contribution in the CECs percentage distribution, i.e., 37%, but in this case emtricitabine had a lower (13%) contribution, which, as mentioned above, could trace back to pharmacodynamics, metabolism, and other interactions. 1,7 DXN (13%), ATZ (7%), TMD (6%), CAF (5%), CBZ (4%) and SMX (4%) also contributed to this score, with BTA, TMP, MTQ having a smaller (2%) contribution and ACT, COD, VFX, CTZ, and NAP contributing 1% or less. Finally, the contribution of DCF, MET, and BZE was insignificant (< 0.1%) to the overall CECs percentage distribution (Fig. 2b).

Overall, efavirenz and emtricitabine exhibited the highest percentage contributions in both water matrices, which reflect the high consumption of antiretroviral medication in the catchment area of the examined water bodies. Specifically, different concentrations have been reported in South Africa with values as high as 34,000 ng/L^66^ for efavirenz and 4000 ng/L^56^ for emtricitabine (FTC) (Table 5). These concentrations can be traced back to the very large number of people (around 26 million) that lives with HIV resides in Sub-Saharan Africa^42^ and likely to antiretroviral drugs abuse^64^. For example, in South Africa recreational antiretroviral abuse has been reported in the form of low-grade smoked heroin, particularly among socially vulnerable youth^65^ (Table 5), which is cut (mixed) with HIV drugs and goes by the street name whoonga, or wonga, or nyaopahi, or nyaope. It is believed that efavirenz is crushed and mixed with other products and illicit drugs for whoonga manufacturing^64^, and this could well be a contributing factor to the observed levels and therefore needs to be further examined in future research. The remaining CECs yielded varying contributions, while their levels in freshwater and wastewater are within the range of previous research in South Africa and further afield (Table 5).

**Table 5:** CECs concentrations in water across the globe.

CEC	Country	Water matrix	Concentration	Reference
Efavirenz	Kenya	River water	ND − 560 ng/L	^67^
	South Africa	River water	0–354 ng/L	^43^
	Kenya	Wastewater	100–1020 ng/L	^67^
	South Africa	Wastewater	9–34,000 ng/L	^66^
Caffeine	South Africa	River water	1170–60,530 ng/L	^45^
	China	River water	66–8571 ng/L	^68^
	India	River water	< LOQ – 6490 ng/L	^69^
	India	Surface water	< LOQ – 3360 ng/L	^70^
	Serbia	Surface water	131–5540 ng/L	^71^
	Nigeria	Surface water	< 4–1080 ng/L	^49^
	Nigeria	Groundwater	< 4–166 ng/L	^49^
	USA	Groundwater	< LOQ – 290 ng/L	^72^
	Slovenian	Wastewater	< LOQ – 7630	^73^
	Korea	Wastewater	0–2349 ng/L	^74^
Sulfamethoxazole	Vietnam	Surface water	612–4330 ng/L	^75^
	Kenya	River water	20–38 850 ng/L	^67^
	Kenya	Wastewater	3260–10,140 ng/L	^67^
	Kenya	Groundwater	20–30 ng/L	^67^
	Nigeria	Surface water	< 1–3180 ng/L	^49^
	Nigeria	Groundwater	< 1–64 ng/L	^49^
	Japan	River water	< LOQ – 78 ng/L	^76^
	Canada	Surface water	5–22 ng/L	^77^
	Bolivia	Wastewater	374–1310 ng/L	^78^
	Korea	Wastewater	20–2016 ng/L	^74^;
Trimethoprim	Kenya	River water	30 − 6 950 ng/L	^67^
	Kenya	Wastewater	90 − 72 850 ng/L	^67^
	Kenya	Groundwater	20–60 ng/L	^67^
	Canada	Surface water	140–220 ng/L	^77^
	Switzerland	Surface water	< LOQ – 250 ng/L	^79^
	Nigeria	Surface water	2–388 ng/L	^49^
	Nigeria	Groundwater	< 1–21 ng/L	^49^
	India	River water	64.5–8808	^69^
	India	Wastewater	< LOQ – 1066 ng/L	^80^
	Korea	Wastewater	13–277 ng/L	^74^
Codeine	Nigeria	Surface water	< 2–1780 ng/L	^49^
	Nigeria	Groundwater	< 2–2440 ng/L	^49^
	UK	River water	9–320 ng/L	^81^
	South Africa	River water	< LOQ – 129 ng/L	^46^
	Spain	River water	30–40 ng/L	^82^
Acetaminophen	South Africa	River water	5800–58,700 ng/L	^45^
	Kenya	River water	< LOQ – 16,000 ng/L	^83^
	Nigeria	Surface water	1–12,430 ng/L	^49^
	Nigeria	Groundwater	< 1-188ng/L	^49^
	Mexico	River water	< LOQ – 4460 ng/L	^84^
	Germany	River water	1–1992 ng/L	^85^
	Spain	River water	< LOQ – 872 ng/L	^86^
	Canada	Surface water	609–749 ng/L	^87^
	Canada	Surface water	43–135 ng/L	^88^
	Korea	Wastewater	0–10 234 ng/L	^74^
Emtricitabine	South Africa	River wate	0–13 ng/L	^43^
	South Africa	Wastewater	1–4000 ng/L	^56^
MDMA	South Africa	Wastewater	ND −14.5	^36^
Diclofenac	South Africa	Surface water	< LOQ – 2580 ng/L	^55^
	Kenya	Surface water	ND – 730 ng/L	^67^
	Kenya	Wastewater	30–1 510 ng/L	^67^
	Nigeria	Surface water	< 1–200 ng/L	^49^
	Nigeria	Groundwater	< 1–42 ng/L	^49^
	Nigeria	Surface water	20–270 ng/L	^89^
	Korea	Wastewater	13–243 ng/L	^74^
Naproxen	South Africa	Surface water	< LOQ – 2770 ng/L	^55^
	Nigeria	Surface water	20–1030 ng/L	^89^
	Nigeria	Surface water	< 3–2120 ng/L	^49^
	Nigeria	Groundwater	< 3–17 ng/L	^49^
	Sweeden	surface water	13–87 ng/L	^90^
	Poland	River water	12–76 ng/L	^91^
	Korea	Wastewater	37–5033 ng/L	^74^
Tramadol	Nigeria	Surface water	< 2–852 ng/L	^49^
	Nigeria	Groundwater	< 2–883 ng/L	^49^
Carbamazepine	Greece	River water	1–325 ng/L	^84^
	China	River water	< LOQ – 146 ng/L	^92^
	Switzerland	Lake water	1–40 ng/L	^93^
	India	River water	< LOQ – 13 ng/L	^94^
	Kenya	River water	ND − 350 ng/L	^67^
	Nigeria	Surface water	< 1–342 ng/L	^49^
	Nigeria	Groundwater	< 1–50 ng/L	^49^
	Kenya	Wastewater	ND – 430 ng/L	^67^
	Italy	Wastewater	< LOQ – 291 ng/L	^95^
	Korea	Wastewater	40–127 ng/L	^74^
Atrazine	India	River water	< LOQ – 3930 ng/L	^96^
	Canada	River water	< LOQ – 754 ng/L	^97^
	Canada	River water	< LOQ – 666 ng/L	^97^
	China	River water	< LOQ – 180 ng/L	^92^
Benzotriazole	China	River water	1–1732 ng/L	^98^
	India	River water	39–526 ng/L	^99^
	Spain	River water	1080–2856 ng/L	^100^
	Spain	Wastewater	214–1469 ng/L	^100^

### Potential ecotoxicological risk assessment for surface water

To estimate the potential ecotoxicological effects of the measured CECs concentrations, the corresponding risk quotients (RQs) were estimated. Results are shown in Table 6, with four (4) CECs posing a possible risk (RQ > 1). Specifically, the cumulative risk index (sum of RQs) for efavirenz was 60.7, for caffeine 28.8, for atrazine 5.2, and for diclofenac 5.0, with the remaining CECs yieldiedvalues lower than unity (Table 6). The overall total risk, in terms of total environmental pressure, was WWTP-Z > river water > WWTP-B > dam water, with efavirenz values suggesting a high risk across sampling locations, as has also been reported elsewhere^40^. Caffeine also yielded high risk for river/stream water and WWTP-B, similarly to the high risk (12.2) reported by Pires et al.^101^. Furthermore, for diclofenac the RQ values suggested a medium risk across sampling locations, whereas this was also the case for atrazine in WWTP-Z, for caffeine in WWTP-B, and for emtricitabine, emtricitabine, acetaminophen, and caffeine in river water. A low risk was observed for sulfamethoxazole in all sampling locations, contrary to the high risk of sulfamethoxazole (2–4 to 9.49) reported elsewhere^53,102^, while cetirizine and methaqualone in WWTP-B and dam water and atrazine in dam water also yielded low risk. Finally, the RQ values for all remaining CECs suggested an insignificant risk, which is not suprising. For example,  Selak et al.^103^, reported RQ values much lower than 1 for carbamazepine in karst catchment water in Croatia, as was also the case here (insignificant risk).

As such, the identified RQ values further highlight that treated municipal wastewater can be a harbinger for CECs , with associated risks, and that raw water (dam and particularly river/stream water) in the South African setting is largely affected by CECs. Finally, as discussed above, the very high RQ values for efavirenz possibly reflects the HIV/AIDs epidemic crisis in Sub-Sahra Africa and likely antiretroviral abuse for illicit drug (whoonga) manufacturing. This further highlights the need for improved water and wastewater monitoring and management practices, as to avoid the release of CECs in the first place and therefore safeguard biodiversity and human health.

Overall, the identified high temporospatial variations demonstrate the need for long-term CECs measurement campaigns, focusing both on different spatial extents and waterbodies as well as denser temporal resolutions. Yet, in South African, and across the developing world where similar restrictions and barriers persists, CECs measurement campaigns are often hindered by technology and mainly cost barriers, which render the collection of such data difficult to acquire. To this end, knowledge transfer and capacity building, along with the proper financial support should be available in such settings to identify the nature and extent of CECs in water and inform effective policy interventions for curbing CECs before reaching the environment.

**Table 6:** The risk quotients (RQs) of the CECs present WWTP effluents and in river/stream and dam water.

Assessment Factor	PNEC (ng/L)^a^	Risk Quotient and Site (ng/L)^b^						I	
		WWTP-Z		River		WWTP-B		Dam	
MET	9740*	0.0534	I	0.0084	I	0.0013	I	0.0051	I
1,7 DXN	21,400*	0.0102	I	0.0310	I	0.0335	I	0.01164	I
MDMA	30,100*	0	I	0	I	0	I	0	I
BZE	7070*	0.0085	I	0.0020	I	0.0020	I	0.0014	I
COC	2460*	0	I	0	I	0	I	0	I
CAF	100**	2.7400	M	14.9200	H	10.3800	H	0.7400	L
NAP	1700**	0.0500	I	0.0400	I	0.0535	I	0.0124	I
DCF	50**	1.9600	M	1.3400	M	1.7400	M	0	I
ACT	46,000**	0	I	0.0702	I	0.0410	I	0	I
TMP	120,000**	0.0087	I	0.0012	I	0.0018	I	0.0087	I
SMX	600**	0.1417	L	0.3667	L	0.2567	L	0.1533	L
EVZ	200*	30.2750	H	11.0500	H	10.1100	H	9.2300	H
FTC	23,800*	0.0637	I	0.1593	M	0.0759	I	0.0176	I
COD	7190*	0	I	0	I	0	I	0	I
TMD	8650*	0.0151	I	0.0151	I	0.0354	I	0.0310	I
VFX	880**	0.1693	L	0.0261	I	0.0159	I	0.0368	I
CTZ	410*	0.4756	L	0.0342	I	0.0878	I	0.1342	L
CBZ	2000**	0	I	0.0320	I	0.0935	I	0	I
BTA	19,000**	0.0055	I	0.0047	I	0.0027	I	0.0042	I
ATZ	600**	4.7733	M	0.0217	I	0.0817	I	0.3227	L
MTQ	720*	0.1542	L	0.0069	I	0.0917	I	0.1403	L
ΣRQs sampling points		40.4286		28.1709		23.0166		10.7151	

^a^ The Predicted No-Effect Concentrations (PNEC) values were extracted from the Norman Ecotoxicology Database and P-PNEC is Provisional Predicted No-Effect Concentrations.

* P-PNECpred.

** PNECfw lt.

Concentrations; pred (Prediction); fw (freshwater) and lt (less than).

^b^ Data show risk quotients for each site (left) and risk level (right) as Insignificant (I), Low (L), Medium (M) or High (H).

### Conclusions and recommendations

This study assessed the occurrence and temporal (intra-seasonal, seasonal, and intra-annual) variations of the contaminants of emerging concern (CEC) or emerging contaminants (EC) in dam water. River/stream water and effluents from two municipal wastewater plants (WWTP B and Z), in the greater area of Pretoria, in South Africa, were also sampled. In total 21 CECs were monitored and for 19 of them levels in the order of few ng/L up to 6  µg/L were identified. In river water, low concentrations for some PhACs were identified (e.g., codeine was not detected while methaqualone’s average concentration was just 5.3 ± 0.3 ng/L), but very high for others (e.g., acetaminophen (3227.8 ± 113.5 ng/L), efavirenz (2210.2 ± 248.5 ng/L), and emtricitabine (3790.7 ± 170.8 ng/L)). CECs concentrations greatly varied between the two examined WWTPs, suggesting a high spatial (and likely temporal, at the hours scale) variability across similar water matrices, since both plants are nearby and follow similar treatment practices. However, large variations when measuring CECs are to be expected. For example, the concentration of atrazine, a widely used herbicide in South Africa^43^, in WWTP-Z (2863.8 ± 60.5 ng/L) was two orders of magnitude higher than the one of WWTP-B (48.8 ± 3.2 ng/L). Furthermore, in WWTP-Z the mean concentration of efavirenz was nearly thrice than in WWTP-B and in both cases high emtricitabine concentrations (1805.5 ± 53.0 ng/L ng/L in WWTP-B and 1515.3 ± 20.9 ng/L in WWTP-Z) were measured. Finally, in dam water high efavirenz and emtricitabine concentrations were also observed, which, reflect the AIDS crisis in South Africa and Sub-Sahara Africa and likely HIV medication abuse.

The monthly measurements suggested high intra-seasonal (coefficient of variation (COV) up to 1.73), seasonal (COV up to 1.66), and intra-annual (COV up to 2.15) variations, which implies that long-term measurements campaigns, at least on a seasonal scale, are required to obtain meaningful results for CECs occurrence and distribution. In the field survey where river/stream water and municipal wastewater effluents were also sampled it was identified that, overall, river water appears to be more affected by CECs compared to dam water, whereas municipal wastewater effluents (treated wastewater) could be a harbinger for CECs.

Regarding the potential risks that the release of these CECs poses, a deterministic approach was followed, and the risk quotients (RQs) were estimated. Efavirenz, caffeine, atrazine, and diclofenac were identified as high risk, particularly for efavirenz, across sampling locations . This was also the caseand for caffeine in river water and in WWTP-B. A medium risk was identified for diclofenac across the examined sampling locations, whereas this was also the case for caffeine in WWTP-B, emtricitabine, acetaminophen, and caffeine in river water, and atrazine in WWTP-Z. A low risk was observed for sulfamethoxazole across sampling locations, while this was the case for cetirizine and methaqualone in WWTP-B and dam water and atrazine in dam water. Finally, the remaining RQ values for all examined CECs suggested an insignificant risk.

Overall, the identified high temporospatial variations demonstrate the need for long-term CECs measurement campaigns, focusing both on different spatial extents and waterbodies as well as denser temporal resolutions. Yet, in South African, and across the developing world where similar restrictions and barriers persists, CECs measurement campaigns could be hindered by technology and mainly cost barriers, which render CECs measurements difficult to acquire. To this end, knowledge transfer and capacity building, along with the proper financial support should be available in such settings to identify the nature and extent of CECs in water and inform effective policy interventions for curbing CECs before reaching the environment.

A main limitation of this study is the limited number of CECs that were evaluated, given that there are hundreds of CECs that have been reported in freshwater and wastewater. Therefore, future research should extend the scope of the analysis to quantify a larger number of CECs, assess their toxicological effects and their remediation using non-target and targeted analytical approach. Finally, current standards and guidelines typically do not regulate CECs, and even they do, significant variations in their limits exist in different regions. Therefore, further research should focus on shedding light on the presence and effects of CECs in water effluents and identify their environmental risk, thereby informing their effective regulation.

## Supplementary Information

Below is the link to the electronic supplementary material.

Supplementary Material 1

## Data Availability

The data can be found in the supplementary material file.
